# Genotype-dependency of butyrate efficacy in children with congenital chloride diarrhea

**DOI:** 10.1186/1750-1172-8-194

**Published:** 2013-12-19

**Authors:** Roberto Berni Canani, Gianluca Terrin, Ausilia Elce, Vincenza Pezzella, Peter Heinz-Erian, Annalisa Pedrolli, Chiara Centenari, Felice Amato, Rossella Tomaiuolo, Antonio Calignano, Riccardo Troncone, Giuseppe Castaldo

**Affiliations:** 1 Department of Translational Medical Science - Pediatric Section, and European Laboratory for the Investigation of Food Induced Diseases, University of Naples, “Federico II” Via S. Pansini, 5 80131 Naples, Italy; 2 European Laboratory for the Investigation of Food Induced Diseases, University of Naples, Federico II, Naples, Italy; 3 Department of Gynecology-Obstetrics and Perinatal Medicine, University of Rome, “La Sapienza”, Rome, Italy; 4 Department of Biochemistry and Medical Biotechnology, University of Naples, Federico II, Naples, Italy; 5 Department of Pediatrics, Medical University, Innsbruck, Austria; 6 Department of Pediatrics, Trento Hospital, Trento, Italy; 7 Department of Pharmacology, University of Naples, Federico II, Naples, Italy; 8 Italy CEINGE–Biotecnologie Avanzate, Naples, Italy

**Keywords:** SLC26A3, SLC26A6, DRA, Mutations, Short chain fatty acids, Pediatrics, Children

## Abstract

**Background:**

Congenital chloride diarrhea (CLD) is an autosomal recessive disorder characterized by life-long, severe diarrhea with intestinal Cl^-^ malabsorption. It results from a reduced activity of the down regulated in adenoma exchanger (DRA), due to mutations in the solute carrier family 26, member 3 (*SLC26A3*) gene. Currently available therapies are not able to limit the severity of diarrhea in CLD. Conflicting results have been reported on the therapeutic efficacy of oral butyrate.

**Methods:**

We investigated the effect of oral butyrate (100 mg/kg/day) in seven CLD children with different *SLC26A3* genotypes. Nasal epithelial cells were obtained to assess the effect of butyrate on the expression of the two main Cl^-^ transporters: DRA and putative anion transporter-1 (PAT-1).

**Results:**

A variable clinical response to butyrate was observed regarding the stool pattern and fecal ion loss. The best response was observed in subjects with missense and deletion mutations. Variable response to butyrate was also observed on *SLC26A3* (DRA) and *SLC26A6* (PAT1) gene expression in nasal epithelial cells of CLD patients.

**Conclusions:**

We demonstrate a genotype-dependency for butyrate therapeutic efficacy in CLD. The effect of butyrate is related in part on a different modulation of the expression of the two main apical membrane Cl^-^ exchangers of epithelial cells, members of the SLC26 anion family.

**Trial registration:**

Australian New Zealand Clinical trial Registry ACTRN12613000450718.

## Introduction

Congenital chloride diarrhea (CLD-OMIM 214700) is an autosomal recessive disorder characterized by life-long, severe diarrhea with intestinal Cl^-^ malabsorption. It results from a reduced activity of the down-regulated in adenoma exchanger (DRA), due to mutations in the solute carrier family 26, member 3 (*SLC26A3*) gene [1-3]. In humans, *SLC26A3* encodes for a 764-amino acid protein and is located on chromosome 7 in a head-to-tail arrangement with *SLC26A4* (pendrin), indicating ancient gene duplication [1-3]. Over 50 different *SLC26A3* mutations, including founder mutations in Finland, Poland, Saudi Arabia and Kuwait populations, have been identified in CLD patients [4]. Such mutations are heterogeneous (mainly missense, insertion/deletion, nonsense and splicing), spread all over the *SLC26A3* gene, and have a different impact on the expression and the activity of DRA [5,6]. Although no genotype-phenotype correlation attributed to different SLC26A3 mutations has been noted, the overall clinical picture and outcome of CLD patients range from severe neonatal disease, with life threatening hypoelectrolytemia and dehydration, to a relatively mild chronic form, which may remain undiagnosed for long time [7-10]. Increasing evidences suggest the importance of early diagnosis and treatment, and of other undefined environmental factors, as modulators of the prognosis and clinical severity of CLD [7-11]. In patients with CLD, supplementation therapy with a combination of Cl^-^ salts (NaCl and KCl) is essential in preventing episodes of dehydration that could result in mental and psychomotor impairment, and in chronic contraction of the intravascular space that could lead to renal dysfunction and gout [7,11]. Unfortunately, this therapy is unable to limit the severity of diarrhea, as for other therapeutic approaches, such as omeprazole, acetazolamide and cholestyramine [12-15].

The role of the amylase-resistant starch has been increasingly recognized for the management of diarrheal diseases [16,17]. Dietary fibres are fermented by gut microbiota into short-chain fatty acids (SCFAs), including acetate, propionate, and butyrate [18-20]. Butyrate exerts a powerful pro-absorptive stimulus on intestinal NaCl transport and an anti-secretory effect on Cl^-^ secretion [2,19,20]. In a child affected by CLD, we demonstrated the therapeutic efficacy of oral butyrate, showing a progressive reduction to normal values in the number of bowel movements and stool volume, an improvement in stool consistency, and a reduction of fecal incontinence episodes. A reduction of fecal electrolyte and persistency of normal serum electrolyte concentrations were also demonstrated [18]. Subsequently, Wedenoja et al. evidenced different results in five CLD patients homozygous for a frameshift mutation [21]. These findings suggest that the variable response to butyrate could depend, at least in part, on different *SLC26A3* genotype.

The two main transporters involved in Cl^-^ absorption at intestinal level are DRA and putative anion transporter 1 (PAT-1), encoded by *SLC26A6* gene [22]. It has been demonstrated that butyrate is able to regulate DRA gene expression in intestinal epithelial cells [22], but the possible effect of butyrate on *SLC26A3* and *SLC26A6* expression in CLD patients is still unknown.

In this study we evaluated the therapeutic effect of butyrate in children affected by CLD with different *SLC26A3* genotype through a clinical trial and an *in vitro* investigation.

## Methods

### Clinical trial

#### Ethics

The study protocol was approved by the Ethics Committee of the University of Naples Federico II (n. 3469/07) and by the Italian Agency for Drugs (AIFA), and it was registered in the Australian New Zealand Clinical trial Registry (ACTRN12613000450718). All authors had access to the study data and had reviewed and approved the final manuscript.

### Population

The Pediatric Gastroenterology Unit at the University of Naples “Federico II” is an International Reference Center for patients with CLD, and served as Coordinator Center of this study. From 2005 to 2010, 35 cases of suspected CLD were referred to the Center, and a definitive diagnosis of CLD was obtained in 25 patients with different ethnicity. Demographic, clinical and laboratory data of all CLD patients were collected in a dedicated data-base. All subjects included in this database were invited to participate in the study with the aim to evaluate at least one patient for each of main mutations (missense, deletion, nonsense and splicing). The physicians of all Centers received by E mail the protocol and any request of information was satisfied by a direct contact with the Coordinator Center. Exclusion criteria were: severe dehydration; concomitant presence of infections; concomitant other chronic diseases; renal insufficiency; use of probiotics/prebiotics, non-steroideal anti-inflammatory drugs (NSAIDs), or antibiotics in the last 4 weeks.

### Genotype definition of children enrolled into the clinical trial

Molecular analysis was performed in the laboratory of CEINGE-Biotecnologie Avanzate , the reference Center for molecular diagnosis of inherited diseases in Campania region (about 6 million of inhabitants), located in southern Italy. DNA was extracted from an EDTA blood sample with the Nucleon BACC2 kit (Amersham Biosciences, USA). The primers used are reported elsewhere [23]. The touchdown PCR protocol that enables co-amplification of all exons under the same PCR conditions is available on request. Sequencing analysis was carried out on both strands with an automated procedure (3100 Genetic Analyzer, Applied Biosystem). All PCR fragments were sequenced with the primers used for PCR. Furthermore, we used the Expand Long Template PCR System (Roche, Germany) to verify deletion extension in patient bearing c.2008-151_2061 + 1546 del mutation. We used the forward primer of exon 17 and the reverse primer of exon 19 [23], both known to be intact in exon-specific assays. The expected fragment is about 6300 bp. The PCR conditions are available on request.

### Intervention

We used a commercially available sodium butyrate formulation (SOBUTIR®, Promefarm, Milan, Italy). Butyrate was administered orally at 100 mg/kg/day, divided in 2 doses, with a maximal dosage of 4 g/day, for 1 week. Number of tablets of sodium butyrate (1 gr/tablet) consumed by the child during the trial were reported in a specific form by the parents. A good compliance was considered the intake of at least 80% of the prescribed doses. Parents of the enrolled children were advised to avoid co-administration of other treatments, including anti-diarrheal drugs, antibiotics, prebiotics or probiotics during the trial. Children continued their normal diet during the study period. Throughout the study period, all CLD subjects were examined as outpatients and they had free access to the services of referred hospitals.

### Trial design and data collection

This was an open trial on subjects with a confirmed diagnosis of CLD. The purposes and the modalities of the study were illustrated to the parents during the first visit (Visit 1), and a written informed consent was obtained from parents or tutors of each enrolled patient. Baseline clinical and laboratory data were collected during the week before butyrate treatment, and were considered representative of the usual pattern of each enrolled patient. In particular, the parents of each patient were instructed to record daily in a specific clinical chart: number of bowel movements, fecal volume, stool consistency (using a scoring system: normal = 0, loose = 1, semi-liquid = 2, liquid =3), and presence of incontinence. At the end of the baseline week of observation, the clinical chart of each child was collected and the patient was re-evaluated (Visit 2). A full clinical evaluation was performed and serum, fecal and urinary ion concentrations were determined, together with serum pH, renin and aldosterone values, as previously described [18]. Fecal electrolyte concentrations were measured on stool samples collected daily during the last 3 days of observational period. The parents of each enrolled subject received a written prescription about the modalities of butyrate administration for 1 week associated with oral NaCl/KCl supplementation, as previously described [18]. In the last 3 days of treatment we collected daily a fecal sample to study the effect of butyrate on fecal Na^+^ and Cl^-^ concentration. At the end of treatment with butyrate, the patients were re-evaluated (Visit 3), and serum, urinary and fecal electrolyte concentrations were measured again. Primary outcome of the study was the reduction of Cl^-^ and Na^+^ fecal losses induced by butyrate therapy.

### *In vitro* study

#### Ex-vivo epithelial cell collection by nasal brushing

Nasal brushing was performed using an endo-brush at the level of the inferior turbinate without anesthetic procedures.

### Epithelial cell culture

The sample obtained from each nostril was immediately conserved in a 15 mL tube containing 2.5 mL of RPMI 1640 medium, complemented with 3% ampicillin. Cells were placed on Eppendorf Thermomixer, in agitation at 300 rpm for one hour. Once removed the brush from every sample, cells were centrifuged at 931 Xg (2000 rpm) for 20 minutes, supernatant was discarded and cell pellet was treated with 150 μL of Trypsin-Versene (EDTA) solution (Lonza, SW) for 4 minutes at 37°C, in order to disaggregate possible cell clusters. Trypsin was inactivated by adding 3 mL of serum-free Bronchial Epithelial cell Growth Medium (BEGM Clonetics, USA). After centrifugation at 2000 rpm for 10 minutes, cells were placed in CELL + T 25 flasks (Sarstedt Ltd, UK). At confluence of 60%, cells were passed in new flasks after count using Invitrogen Cell Countess (Invitrogen, UK). Trypan blue exclusion test was used in order to establish total viable cells number and percentage of viability.

### Effect of butyrate on epithelial cells

At the confluence of >80%, cells were treated with 5 mM of sodium butyrate for 24 hours. RNA was extracted with TRIZol method (Invitrogen, UK). Total RNA amount was quantified with Nanodrop 1000 spectrophotometer (Thermo Fisher Scientific, UK). One microgram of total RNA was retro-transcribed with Quantitect Reverse Transcription kit (Qiagen, Germany), cDNA was diluted for downstream applications like quantitative real-time PCR analysis. Expression levels of either SLC26A3 and SLC26A6 from treated and untreated cells were measured by semi-quantitative real-time PCR on LightCycler 480 Real-Time PCR System (Roche, Germany) with Taqman probe chemistry (experiments were performed in replicate using also SYbr Green chemistry). Results were normalized for housekeeping glyceraldheyde 3-phosphate dehydrogenase (GAPD) gene. Levels of SLC26A3 and SLC26A6 expression before and after treatment were compared in order to establish changes determined by butyrate exposure. Calculation of relative gene expression was performed according to Pfaffl et al. [24], and the expression was calculated using the formula of relative gene expression with DDCt method (where DDCt corresponds to the increase in the threshold cycle of the target gene with respect to the increase in the threshold cycle of the housekeeping gene). Hence, the final quantification value for each condition indicated the relative change of gene expression in the target gene compared to the control, for each sample.

### Statistical analysis

The Kolmogorov-Smirnov test was used to determine whether variables were normally distributed. The chi-square test was applied for categorical variables, and for continuous variables, differences between groups were analyzed by Mann- Whitney U test, and Kruskal-Wallis H test. A multivariate analysis was performed to evaluate if the effects of butyrate may depend by clinical or genetic factors. All analyses were conducted on an intention-to-treat (ITT) basis. Statistical analysis was carried out by the SPSS software for Windows 16.0 and by StatDirect 1.7.

## Results

### Clinical trial

**Figure 1:**
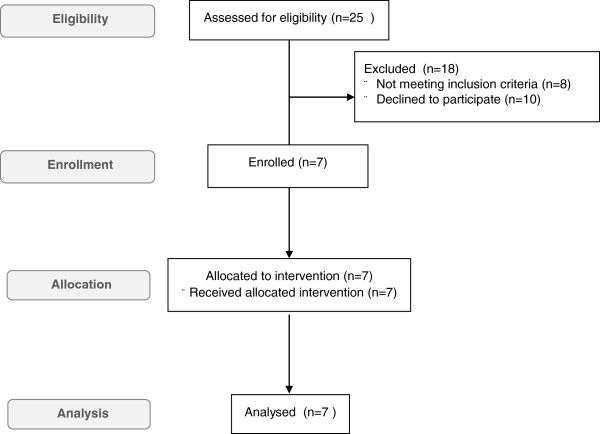
Flow diagram of the study (according to CONSORT guidelines).

Enrollment of patients was performed from January 2010 to December 2012. Out of 25 eligible children with CLD, 18 subjects were excluded (8 not meeting inclusion criteria, 10 declined to participate because different Ethics Committee regulatory procedures and logistic difficulties), 7 were enrolled in the study (Figure 1). All patients showed a typical clinical picture of CLD with early onset diarrhea. Three subjects received a late diagnosis.

SLC26A3 genotype was defined for each subjects. Patients 1, 3 and 7 presented previously unreported mutations. Patients 1 and 2 had missense mutations and the protein was expressed on cell membrane. Patient 3 was homozygous for a large deletion that caused the synthesis of a truncated protein and some amount of the protein was present at membrane level. Patients 4 to 6 were homozygous for nonsense mutations with no protein expression at membrane level. Patient 7 was homozygous for a splicing mutation that causes the synthesis of an aberrant mRNA, with no protein expression at membrane level. The genotype and main demographic features of study subjects are reported in Table 1.

**Table 1:** Main demographic characteristics and genotype of patients with congenital chloride diarrhea

**Patient**	**Age at diagnosis**	**Age the enrollment**	**Sex**	**Ethnic origin**	**Body weight (kg)**	***SLC26A3 *** **genotype**	**Type of mutation**
1	6.5 y	16 y	M	Caucasian	70	c.1484A > C*	Missense
						c.1640C > A*	
2	4 m	3 y	M	Caucasian	22	c.386C > T	Missense
						c.386C > T	
3	6 y	12 y	M	Caucasian	32	c.1008-151_2061 + 1546del*	Deletion
						c.1008-151_2061 + 1546del*	
4	3 m	18 y	F	Caucasian	58	c.2132 T > G	Nonsense
						c.2132 T > G	
5	1 m	18 y	M	African	55	c.559G > T	Nonsense
						c.559G > T	
6	1 m	15 y	M	African	57	c.559G > T	Nonsense
						c.559G > T	
7	10 m	1.5 y	M	Caucasian	10	c.1408-G > C*	Splicing
						c.1408-G > C*	

*Novel mutations.

**Figure 2:**
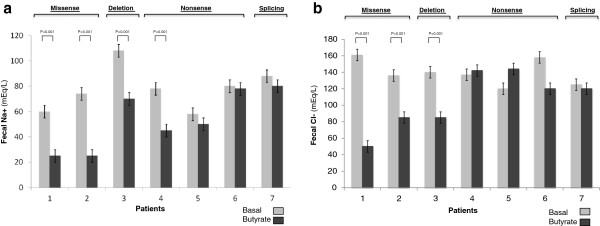
**Fecal sodium (a) and chloride concentration (b) in children with congenital chloride diarrhea treated with butyrate.** Box and bar represent median and min-max range, respectively.

All patients were evaluated in stable clinical conditions. Overall, butyrate therapy induced a reduction of Cl^-^ (136 mmol/l, IQR 13 *vs* 120 mmol/l, IQR 42; p < 0.001) and Na^+^ (78 mmol/l, IQR 29 *vs* 50 mmol/l, IQR 49; p = 0.002) fecal losses in CLD patients, but a variable response was observed in children with different *SLC26A3* genotype. The more evident reduction of fecal ion losses was observed in patients with missense and deletion mutations (Figure 2).

A variable clinical response was also observed on stool pattern in CLD patients with different mutations. Clinical response (defined by a concomitant significant reduction of ≥2 variables) was observed in patients with missense and deletion mutations (Table 2). A reduction of incontinence episodes was observed in patients 1, 2 and 3. The effect of butyrate on stool pattern became evident within the first 48 hours and remained stable during the following days of treatment.

**Table 2:** Effects of butyrate on daily stool pattern in patients with congenital chloride diarrhea

**Patient**	**Bowel movements**			**Stool volume (ml)**			**Stool consistency (score)**		
	**Basal**	**On Butyrate**	***p***	**Basal**	**On Butyrate**	***p***	**Basal**	**On Butyrate**	***p***
1	4 (3)	2 (1)	0.003	2100 (500)	1600 (450)	0.047	3 (2)	2 (1)	0.379
	(3–6)	(2–3)		(1800–2300)	(1400–1850)		(1–3)	(2–2)	
2	4 (2)	3 (1)	0.147	1400 (450)	1200 (300)	0.046	3 (0)	2 (1)	0.023
	(3–5)	(3–4)		(1200–1650)	(1000–1300)		(3–3)	(2–3)	
3	3 (2)	2 (0)	0.021	1500 (200)	900 (400)	0.015	3 (0)	2 (1)	0.107
	(2–4)	(2–2)		(1400–1600)	(800–1200)		(3–3)	(2–3)	
4	6 (2)	6 (1)	0.892	2000 (400)	1500 (200)	0.053	3 (0)	2 (1)	0.037
	(5–7)	(6–7)		(1800–2200)	(1500–1700)		(3–3)	(2–3)	
5	2 (2–3)	2 (1)	0.263	1200 (200)	1100 (300)	0.261	2 (1)	3 (1)	0.606
	(2–3)	(1–2)		(1100–1300)	(900–1200)		(2–3)	(2–3)	
6	2 (2)	1 (0)	0.238	900 (200)	800 (400)	0.435	2 (1)	2 (1)	0.872
	(0–2)	(1–1)		(800–1000)	(700–1100)		(1–2)	(1–2)	
7	3 (1)	4 (1)	0.299	1000 (200)	900 (200)	0.289	2 (1)	2 (0)	0.254
	(3–4)	(3–4)		(900–1100)	(800–1000)		(1–2)	(2–2)	

Note. Data are expressed as median (interquartile range). Stool consistency score: normal = 0, loose = 1; semi-liquid = 2; liquid = 3.

The multivariate analysis revealed that only the genotype significantly (p = 0.008) influence the response to butyrate treatment (i.e. missense and deletion mutations that allow the expression of DRA at membrane level). The study procedures and oral butyrate treatment were well accepted by the patients. Serum and urinary electrolyte concentrations, and serum pH, renin and aldosterone levels remained stable within normal ranges during the study period. In Table 3 were summarized the main results of butyrate therapy according to the type of mutations observed in CLD patients.

**Table 3:** Effects of butyrate on fecal electrolytes loss and on stool pattern, according to variation of genotype in patients with congenital chloride diarrhea

	**Genotype**		**Response to butyrate**	
**Patient**	***SLC26A3 *** **genotype**	**Type of mutation**	**Fecal Cl**^**- **^**loss**	Stool Pattern*
1	c.1484A > C*	Missense	Reduced	Improved
	c.1640C > A*			
2	c.386C > T	Missense	Reduced	Improved
	c.386C > T			
3	c.1008-151_2061 + 1546del*	Deletion	Reduced	Improved
	c.1008-151_2061 + 1546del*			
4	c.2132 T > G	Nonsense	Unchanged	Unchanged
	c.2132 T > G			
5	c.559G > T	Nonsense	Unchanged	Unchanged
	c.559G > T			
6	c.559G > T	Nonsense	Unchanged	Unchanged
	c.559G > T			
7	c.1408-G > C*	Splicing	Unchanged	Unchanged
	c.1408-G > C*			

*defined as a significant reduction of a least two out of three parameters (bowel movements, stool volume, stool consistency).

### *In vitro* study

**Figure 3:**
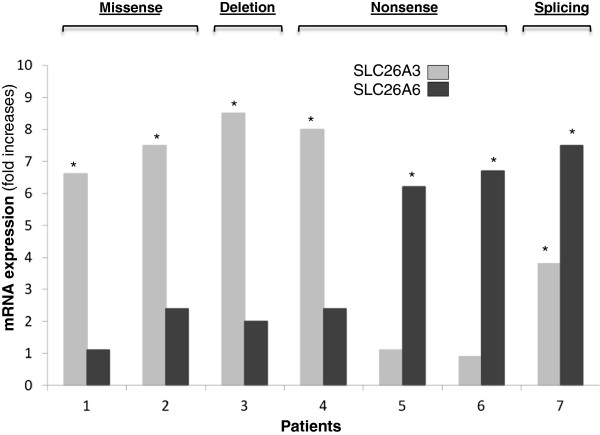
***SLC26A3/DRA *mRNA and *SLC26A6/PAT-1 *mRNA expression in epithelial cells of children enrolled into the trial before and after *in vitro *stimulation with butyrate.** *p < 0.05 vs. basal.

A variable response to butyrate was observed on *SLC26A3* mRNA expression in epithelial nasal cell culture. Butyrate was able to increase the expression of SLC26A3 gene in 5 out of 7 CLD patients (i.e., patients 1, 2, 3, 4 e 7). In two cases (i.e., patients 5 and 6, both homozygous for the G187X nonsense mutation) butyrate significantly inhibited *SLC26A3* expression (Figure 3). The *SLC26A6* mRNA expression resulted significantly increased by butyrate in epithelial nasal cells from 5 out of 7 CLD patients, i.e., cases 2, 4, 5, 6 and 7, while it was significantly reduced in one case (i.e., patient 1) and remained unchanged in patient 3 (Figure 3).

## Discussion

This is the first study exploring the efficacy of butyrate in a population of CLD subjects with different mutations in *SLC26A3* gene. We confirm the efficacy of butyrate on intestinal ion transport in a subset of CLD patients and we demonstrate that the clinical effect of butyrate is at least in part dependent on genotype. A full response to butyrate (defined by a concomitant significant reduction of Na^+^ and Cl^-^ fecal losses and improvement in stool pattern) was observed only in patients with missense and deletion mutations. On the contrary, a partial response was observed in patients with nonsense or splicing mutations.

It has been previously demonstrated that butyrate is able to modulate transepithelial ion transport through at least 2 mechanisms: i) stimulation of Na^+^/H^+^ exchangers 2 (NHE2) and 3 (NHE3) activity; ii) inhibition of Cl^-^ secretion by limiting the action of the co-transporter Na-K-2Cl (encoded by *NKCC1*) on enterocyte baso-lateral membrane [19]. It has been shown that butyrate stimulates DRA expression in LS174T colonic cells [25], whenever data on a possible activity on PAT-1 gene expression were unavailable. Now we demonstrate that, in CLD patients, butyrate is able to modulate the expression of the two main intestinal Cl^-^ transporters: DRA and PAT1. The effect elicited on these two transporters could be considered a third potential mechanism of action, and it could be involved in the genotype-dependency of butyrate effect in CLD patients. The effect of butyrate on these two transporters seems to be different according to the type of SLC26A3 mutation. Patients with non-sense or splicing mutations showed a lower response in SLC26A3, but a more pronounced increase in SLC26A6 mRNA expression; whereas patients with missense or deletion mutation showed a more pronounced increase in SLC26A3 expression and a lower effects on SLC26A6 mRNA expression in nasal epithelial cell culture. Interestingly, a 3-fold up-regulation of PAT-1 expression was detected in DRA-knockout mice [26]. Altogether, these findings suggest that the up-regulation of PAT-1 in CLD patients may play a compensatory role in electrolyte homeostasis. DRA has been shown to be the major apical Cl^-^ absorbing isoform in the colon and ileum able to regulate a large amount of water daily [25]. Additionally, studies have shown that DRA-knockout mice show reduction in apical Cl^-^/HCO_3_ exchange activity and exhibit diarrheal phenotype with significant increased Cl^-^ and water stool content [25]. On the contrary, although PAT-1 is involved in Cl^-^ transport, unlike DRA it is not directly coupled to the water movements demonstrated by PAT-1-knockout mice showing a reduction in Cl^-^ absorption, but not exhibiting a diarrheal phenotype [26,27]. Thus it is possible to hypothesize that the effect induced by butyrate on fecal ions loss is due at least in part by a regulatory action on PAT-1 expression.

The variable butyrate effect in CLD patients seems to be influenced by SLC26A3 genotype. However, the different diarrhea-reducing responses of butyrate observed among patients with similar mutations strongly suggests the existence of other still unidentified regulatory elements. The hypothesis that butyrate may display a large pattern of biochemical effects on intestinal ion channels with a strong inter-individual variability is also supported by a study on 5 CLD patients, all homozygous for a deletion mutation, showing a variable clinical response to this treatment [21].

We feel that the evidence of an improved clinical outcome by butyrate at least in a subset of CLD patients is of potential importance either for the therapeutic management and for the interpretation of the mechanisms that regulate ion absorption at intestinal level in this condition. The effects of endogenous production of butyrate elicited by different dietary habits could be able to influence the clinical picture in CLD patients with same genotype, as previously reported [19].

## Conclusion

We demonstrate that butyrate may act efficiently on either fecal ion loss, and on the severity of diarrhea in a subset of CLD patients. The activity of butyrate seems to be more complex than expected, depending either on the profile of the SLC26A3 gene mutations, but also on other genes, and our study starts to make light on this network.

## Abbreviations

CLD: Congenital chloride diarrhea; SLC: Solute carrier; PAT-1: Putative anion transporter 1; NSAIDs: Non-steroideal anti-inflammatory drugs; GAPD: Glyceraldheyde 3-phosphate dehydrogenase.

## Competing interests

The research was not funded by any Company. No other conflicts of interested were reported.

## Author’s contribution

BCR, TG, CA, TR and CG designed the study and wrote the first draft of the paper. EA, TR and AF performed *in vitro* study. HEP, PA, CC, PV and TR cared for the patients and participated in the writing of the paper. TG performed data analysis. All authors contributed to the final version of the manuscript and approved the content of the paper.

## References

[B1] Terrin G Tomaiuolo R Passariello A Elce A Amato F Di Costanzo M Castaldo G Canani RB Congenital diarrheal disorders: an updated diagnostic approach Int J Mol Sci 2012 8 4168 4185 22605972 10.3390/ijms13044168 PMC3344208

[B2] Berni Canani R Terrin G Cardillo G Tomaiuolo R Castaldo G Congenital diarrheal disorders: improved understanding of gene defects is leading to advances in intestinal physiology and clinical management J Pediatr Gastroenterol Nutr 2010 8 360 366 20216094 10.1097/MPG.0b013e3181d135ef

[B3] Berni Canani R Terrin G Recent progress in congenital diarrheal disorders Curr Gastroenterol Rep 2011 8 257 264 10.1007/s11894-011-0188-6 21494839

[B4] Hoglund P Auranen M Socha J Popinska K Nazer H Rajaram U Al Sanie A Al-Ghanim M Holmberg C de la Chapelle A Kere J Genetic background of congenital chloride diarrhea in high incidence populations: Finland, Poland, and Saudi Arabia and Kuwait Am J Hum Genet 1998 8 760 768 9718329 10.1086/301998 PMC1377387

[B5] Lechner S Ruemmele FM Zankl A Lausch E Huber WD Mihatsch W Phillips AD Lewindon P Querfeld U Heinz-Erian P Müller T Janecke AR Significance of molecular testing for congenital chloride diarrhea J Pediatr Gastroenterol Nutr 2011 8 48 54 21694535 10.1097/MPG.0b013e31820bc856

[B6] Wedenoja S Pekansaari E Hoglund P Makela S Holmberg C Kere J Update on SLC26A3 mutations in congenital chloride diarrhea Hum Mutat 2011 8 715 722 21394828 10.1002/humu.21498

[B7] Hihnala S Hoglund P Lammi L Kokkonen J Ormala T Holmberg C Long-term clinical outcome in patients with congenital chloride diarrhea J Pediatr Gastroenterol Nut 2006 8 369 375 10.1097/01.mpg.0000214161.37574.9a 16641574

[B8] Lok KH Hung HG Li KK Li KF Szeto ML Congenital chloride diarrhea: a missed diagnosis in an adult patient Am J Gastroenterol 2007 8 1328 1329 17531020 10.1111/j.1572-0241.2007.01146.x

[B9] Passariello A Terrin G Baldassarre ME De Curtis M Paludetto R Berni Canani R Diarrhea in neonatal intensive care unit World J Gastroenterol 2010 8 2664 2668 20518089 10.3748/wjg.v16.i21.2664 PMC2880780

[B10] Salvia G Guarino A Terrin G Cascioli C Paludetto R Indrio F Lega L Fanaro S Stronati M Corvaglia L Tagliabue P De Curtis M Neonatal onset intestinal failure: an Italian Multicenter Study J Pediatr 2008 8 674 676 18589446 10.1016/j.jpeds.2008.05.017

[B11] Wedenoja S Hoglund P Holmberg C Review article: the clinical management of congenital chloride diarrhea Aliment Pharmacol Ther 2010 8 477 485 19912155 10.1111/j.1365-2036.2009.04197.x

[B12] Clark EB Effect of acetazolamide on electrolyte balance in congenital chloridorrhea J Pediatr 1997 8 148 149 10.1016/s0022-3476(77)80468-x 874652

[B13] Aichbichler BW Santa Ana CA Porter JL Zerr CH Fordtran JS Proton-pump inhibition of gastric chloride secretion in congenital chloridorrhea N Engl J Med 1997 8 106 109 8988888 10.1056/NEJM199701093360205

[B14] Laine L Ahnen D McClain C Solcia E Walsh JH Potential gastrointestinal effects of long term acid suppression with proton pump inhibitors Aliment Pharmacol Ther 2000 8 651 668 10848649 10.1046/j.1365-2036.2000.00768.x

[B15] Pieroni KP Bass D Proton pump inhibitor treatment for congenital chloride diarrhea Dig Dis Sci 2011 8 673 676 21127979 10.1007/s10620-010-1491-z

[B16] Topping DL Clifton PM Short-chain fatty acids and human colonic function roles of resistant starch and nonstarch polysaccharides Physiol Rev 2001 8 1031 1064 11427691 10.1152/physrev.2001.81.3.1031

[B17] Raghupathy R Ramakrishna BS Oommen SP Ahmed MS Pryaa G Dziura J Young GP Binder HJ Amylase-resistant starch as adjunct to oral rehydration therapy in children with diarrhea J Pediatr Gastroenterol Nutr 2006 8 362 368 16641573 10.1097/01.mpg.0000214163.83316.41

[B18] Berni Canani R Terrin G Cirillo P Castaldo G Salvatore F Cardillo G Coruzzo A Troncone R Butyrate as an effective treatment of congenital chloride diarrhea Gastroenterology 2004 8 630 634 10.1053/j.gastro.2004.03.071 15300594

[B19] Berni Canani R Di Costanzo M Leone L Pedata M Meli R Calignano A Potential beneficial effects of butyrate in intestinal and extraintestinal diseases World J Gastroenterol 2011 8 1519 1528 10.3748/wjg.v17.i12.1519 PMC3070119 21472114

[B20] Berni Canani R Di Costanzo M Leone L The epigenetic effects of butyrate: potential therapeutic implications for clinical practice Clinical Epigenetics 2012 8 4 22414433 10.1186/1868-7083-4-4 PMC3312834

[B21] Wedenoja S Holmberg C Hoglund P Oral butyrate in treatment of congenital chloride diarrhea Am J Gastroenterol 2008 8 252 254 18184140 10.1111/j.1572-0241.2007.01562_14.x

[B22] Malakooti J Saksena S Gill RK Dudeja PK Transcriptional regulation of the intestinal luminal Na and Cl transporters Biochem J 2011 8 313 325 21726200 10.1042/BJ20102062 PMC3377368

[B23] Haila S Höglund P Scherer SW Lee JR Kristo P Coyle B Trembath R Holmberg C de la Chapelle A Kere J Genomic structure of the human congenital chloride diarrhea (CLD) gene Gene 1998 8 1–2 87 93 9729124 10.1016/s0378-1119(98)00261-3

[B24] Pfaffl MW A new mathematical model for relative quantification in real-time RT-PCR Nucleic Acids Res 2001 8 e45 11328886 10.1093/nar/29.9.e45 PMC55695

[B25] Alrefai WA Wen X Jiang W Katz JP Steinbrecher KA Cohen MB Williams IR Dudeja PK Wu GD Molecular cloning and promoter analysis of downregulated in adenoma (DRA) Am J Physiol Gastrointest Liver Physiol 2007 8 G923 G934 17761837 10.1152/ajpgi.00029.2007

[B26] Schweinfest CW Spyropoulos DD Henderson KW Kim J Chapman JM Barone S Worrell RT Wang Z Soleimani M SLC26A3 (dra)-deficient mice display chloride-losing diarrhea, enhanced colonic proliferation, and distinct up-regulation of ion transporters in the colon J Biol Chen 2006 8 37962 37971 10.1074/jbc.M607527200 17001077

[B27] Seidler U Rottinghaus I Hillesheim J Chen M Riederer B Krabbenhöft A Engelhardt R Wiemann M Wang Z Barone S Manns MP Soleimani M Sodium and chloride absorptive defects in the small intestine in SLC26A6 null mice Pflugers Arch 2008 8 757 766 17763866 10.1007/s00424-007-0318-z

